# Gut Microbiome as a Risk Factor for Future CKD

**DOI:** 10.1016/j.ekir.2025.04.007

**Published:** 2025-04-07

**Authors:** Tapio Hellman, Li-Fang Yeo, Joonatan Palmu, Aki Havulinna, Pekka Jousilahti, Ville Laitinen, Katariina Pärnänen, Veikko Salomaa, Leo Lahti, Rob Knight, Teemu Niiranen

**Affiliations:** 1 Kidney Center, Turku University Hospital and University of Turku, Turku, Finland; 2 Division of Medicine, University of Turku, Turku, Finland; 3 Department of Internal Medicine, Turku University Hospital and University of Turku, Turku, Finland; 4 Department of Public Health, Finnish Institute for Health and Welfare, Helsinki, Finland; 5 Institute for Molecular Medicine Finland (FIMM), HiLIFE, University of Helsinki, Helsinki, Finland; 6 Department of Computing, University of Turku, Turku, Finland; 7 Department of Pediatrics, University of California San Diego, La Jolla, California, USA; 8 Center for Microbiome Innovation, Joan and Irwin Jacobs School of Engineering, University of California San Diego, La Jolla, California, USA; 9 Department of Bioengineering, University of California San Diego, La Jolla, California, USA; 10 Department of Computer Science and Engineering, University of California San Diego, La Jolla, California, USA; 11 Halıcıoğlu Data Science Institute, University of California, San Diego, La Jolla, California, USA

**Keywords:** clinical study, chronic kidney disease, microbiology, proteinuria

## Abstract

**Introduction:**

Gut microbiome has been linked with chronic kidney disease (CKD) in several small cross-sectional studies. However, the relationship between baseline gut microbiome and long-term incident CKD remains unknown.

**Methods:**

We performed fecal sampling and measured serum creatinine (SCR) (*N* = 6699) and urine albumin-to-creatinine ratio (UACR) (*N* = 797) in a population-based cohort examined in the year 2002. We assessed the multivariable-adjusted associations of gut metagenome with baseline SCR, baseline UACR, and register-based incident CKD.

**Results:**

The mean age of the participants was 49.5 ± 12.9 years and 45.8% were men. During a median follow-up of 18.6 years, 108 participants developed incident CKD. In prospective analyses, increased baseline gut microbiome alpha diversity was associated with lower risk of incident CKD (hazard ratio per 1 SD: 0.84; 95% confidence interval [CI]: 0.71–0.99; *P* = 0.04). Gut microbial beta diversity and taxa were not related to incident CKD (*P* ≥ 0.09 for all). In cross-sectional analyses, alpha diversity (beta per 1 SD: 1.28; 95% CI: 0.64–1.98; *P* < 0.001) and beta diversity (*P* = 0.002; R^2^ = 0.12%) were associated with SCR, whereas no associations were observed for UACR. In total, 43 significant species-level associations with SCR were observed and 16 negative associations (37.2%) for species belonging to the *Lachnospiraceae* family.

**Conclusion:**

Our results suggest that decreased gut microbial diversity may be related to risk of future CKD and that a potential link between the *Lachnospiraceae* family and desirable kidney health exists. Our results extend previous cross-sectional studies and help to establish the basis for examining gut microbiome as a CKD risk factor.

## Graphical abstract

**Figure:**
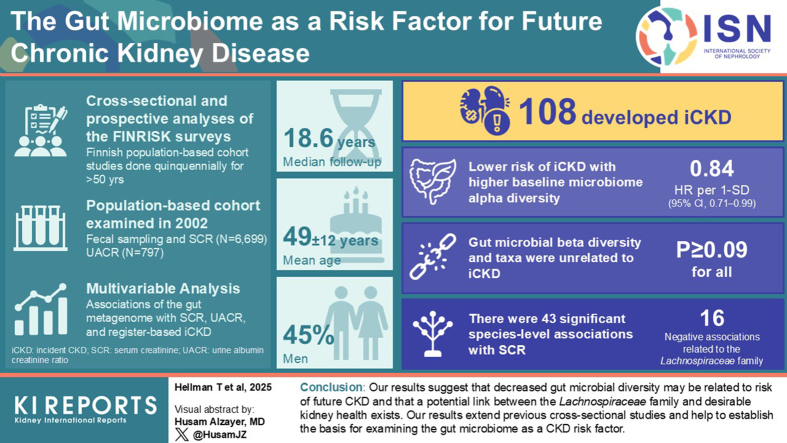

CKD affects approximately 10% of the population worldwide and is a prominent driver for substantial and increasing health care costs globally.^1^ Although classic cardiovascular risk factors such as hypertension and diabetes increase CKD risk, emerging research has shed light on the role of gut microbiome as a potential novel risk factor for CKD occurrence and progression.^2^^,^^3^ Generally, decreased microbial diversity has been associated with CKD in cross-sectional studies, and the overall composition of gut microbiome is distinguishably different in patients with CKD compared with controls with normal kidney function. In addition, several microbial taxa, such as *Blautia* and *Roseburia*, have been linked to current CKD.^2^, ^3^, ^4^, ^5^, ^6^ This relation between gut microbiome and renal function has been referred to as the “gut-kidney axis.”^7^

To date, the associations between gut microbiome and CKD have been mainly examined in cross-sectional case-control studies with limited sample sizes ranging from 24 to 2438 participants.^2^, ^3^, ^4^, ^5^, ^6^ Furthermore, many previous studies have been performed solely using animal models.^7^ Despite these cross-sectional results, the long-term association between gut microbiome and future CKD onset in healthy individuals is unclear. A prospective cohort study design would be optimal to establish a higher level of evidence on the links between gut microbiome and CKD. This approach would clarify the temporal sequence of events (did the exposure precede the outcome?), a common challenge in microbiome-CKD studies, in which the disease and medications treating the disease both modify the microbiome.^8^

To elucidate the relation between gut microbiome and long-term CKD incidence, we collected stool samples and assessed the baseline kidney function in 6699 participants of the population-based FINRISK survey in the year 2002. The participants were then followed-up for incident CKD for a median of 18.6 years, during which 108 participants (2.0%) developed CKD. In addition to assessing cross-sectional links, we investigated the role of gut microbiome as a risk factor for incident CKD.

## Methods

### Study Sample

The FINRISK surveys are population-based cohort studies that have been conducted quinquennially for over 50 years by the Finnish Institute for Health and Welfare.^9^ In 2002, 8799 subjects (65.5%) participated in the FINRISK 2002 study out of the 13,437 invited individuals. The participants were aged 25 to 74 years and originated from 6 separate geographic regions in Finland. Before the baseline examination, all consented study participants completed extensive questionnaires regarding relevant sociodemographic, lifestyle, medicinal, and medical history data. The participants then underwent a health examination, including venous blood sampling, and were given a stool collection kit. In addition, a subsample of 828 participants took part in a 24-hour urine collection after the baseline examination.

The study sample for the cross-sectional analyses for baseline serum creatinine (SCR) (sample 1) included 6699 individuals; for UACR (sample 2), 797 individuals; and for the prospective analyses (sample 3) 6556 individuals (Supplementary Methods).

### Microbiome Sampling and Sequencing

The stool sampling, processing, and microbiome analyses have been described previously.^10^ In brief, stool samples were collected at study baseline at home in 50 ml Falcon tubes and mailed to the Finnish Institute for Health and Welfare overnight and frozen in −20 °C until metagenomic sequencing in 2017. The sequencing was performed at the University of California San Diego according to standard Earth Microbiome Project protocols.^11^ The microbiome analysis was performed with a whole-genome untargeted shallow shotgun metagenomic sequencing against mapped reference databases, as previously described.^10^ Taxonomy was assigned using the Greengenes2 database^12^ and the data was imported in R TreeSummarizedExperiment container^13^ for downstream analysis. Functional pathways abundances were annotated using MetaCyc^14^ database in the HUMAnN pipeline.^15^

### Covariates

Blood pressure was measured in all participants at baseline 3 times by a nurse with a mercury sphygmomanometer. Blood pressure was defined as the mean of all 3 measurements. Body mass index was recorded in kg/m^2^. Use of antihypertensive medications was self-reported. Smoking was denoted as self-reported current daily smoking. Data on relevant diseases (diabetes, heart failure, CKD, and autoimmune disease) were collected from the nationwide Causes-of-Death, Hospital Discharge, and Drug Reimbursement Registers (Supplementary Table S1). The validity of the hospital discharge registers has been previously assessed.^16^

### Outcome Variables

Baseline SCR and UACR were selected as the outcome measures for the cross-sectional analyses (Supplementary Methods) and register-based incident CKD for the prospective analyses (Supplementary Table S1).

### Ethics

The FINRISK 2002 study complies with the Declaration of Helsinki. The Coordinating Ethics Committee of the Helsinki and Uusimaa University Hospital District approved the FINRISK 2002 study. All participants provided written informed consent.

### Statistical Methods

R version 4.3.1 was used for all statistical analyses.^17^ The source code for the analyses is available at https://doi.org/10.5281/zenodo.14327049. Alpha diversity (Shannon index as a measure of mean species diversity as variation and richness in the sample) was defined using species-level data with the R package mia 1.13.31.^18^ The dissimilarity matrix (beta diversity indicating the variation in species abundance profiles between samples) was calculated with supervised ordination technique dbRDA, using Bray-Curtis dissimilarity on compositional microbial species-level abundances. To exclude rare and low-abundance taxa, species-level analyses were limited to the 280 microbial species prevalent in at least 5% of the sample population with a relative abundance > 0.1%, transformed with center log-ratio transformation. The functional MetaCyc pathway abundances were filtered for species-specific pathways prevalent in at least 10% of the sample population. Because of the highly sparse and zero-enriched nature of the pathway data, the counts were dichotomized (present vs. absent), or (inverse-rank normalized for the analyses.

In the cross-sectional analyses, the associations of alpha diversity, microbial taxa, and functional pathways with SCR, estimated glomerular filtration rate (eGFR), and UACR were assessed using linear regression. In the prospective analyses, the associations of alpha diversity, microbial taxa, and functional pathways with incident CKD were assessed using Cox proportional hazards models. As a sensitivity analysis, the association between microbial taxa and incident CKD was assessed with ANCOM-BC2.^19^ The associations of beta diversity with SCR, eGFR, UACR, and incident CKD were assessed using PERMANOVA.

*P*-values were adjusted for multiple testing using false discovery rate (Benjamini–Hochberg correction). False discovery rate *P*-values < 0.05 were deemed statistically significant. All analyses were adjusted for the following: (i) age and sex or (ii) age, sex, baseline SCR, body mass index , diabetes, systolic blood pressure, antihypertensive medication use, smoking, heart failure, and autoimmune disease.

## Results

The baseline characteristics of the study participants are summarized in Table 1. The mean age of the study sample was 49.5 ± 12.9 years and 45.8% were men. The mean SCR, eGFR, and UACR at baseline were 72.3±14.3 μmol/l, 95.7±15 ml/min per 1.73 m^2^, and 1.19±7 mg/mmol, respectively. At baseline, 111 (1.66%) participants had eGFR < 60 ml/min per 1.73 m^2^ and 27 (3.39%) had UACR ≥ 3 mg/mmol.

**Table 1:** Characteristics of study participants

Characteristics	Cross-sectional sample	Prospective sample		
		Overall	Incident CKD	No Incident CKD
*n*	6699	6556	108	6448
Age, yr (SD)	49.5 (12.9)	49.2 (12.8)	59.6 (11.0%)	49.1 (12.7%)
Men	3068 (45.8%)	3007 (45.9%)	73 (67.6%)	2934 (45.5%)
BMI (SD)	27.0 (4.7)	27 (4.6)	29.2 (5.7)	26.9 (4.6)
Diabetes	294 (4.4%)	276 (4.2%)	15 (13.9%)	261 (4.0%)
Systolic BP, mm Hg (SD)	136 (20.2)	136 (20.0)	147 (20.8)	135 (20.0)
Antihypertensive medication	1045 (15.6%)	966 (14.7%)	39 (36.1%)	927 (14.4%)
Heart failure	77 (1.1%)	65 (1.0%)	10 (9.3%)	55 (0.9%)
Follow-up time, yr (SD)	18.5 (3.8)	18.6 (3.5)	14.3 (3.6)	18.6 (3.5)
Smoking	1573 (23.5%)	1545 (23.6%)	20 (18.5%)	1525 (23.7%)
Autoimmune disease	133 (2.0%)	125 (1.9%)	3 (2.8%)	122 (1.9%)
Serum creatinine, μmol/l (SD)	72.3 (14.3)	71.5 (11.9)	79.5 (13.2)	71.4 (11.8)
eGFR, ml/min per 1.73 m^2^ (SD)	95.7 (15.0)	96.4 (13.9)	85.2 (14.4)	96.6 (13.8)
UACR, (mg/mmol) (SD)	1.19 (7.0)	0.66 (0.4)	0.51 (0.2)	0.66 (0.4)
Shannon diversity (SD)	4.11 (0.4)	4.11 (0.4)	4.01 (0.5)	4.11 (0.4)

BMI, body mass index; BP, blood pressure; CKD, chronic kidney disease, eGFR, estimated glomerular filtration rate; UACR, urinary albumin-creatinine ratio.

### Cross-Sectional Analyses

**Figure 1:**
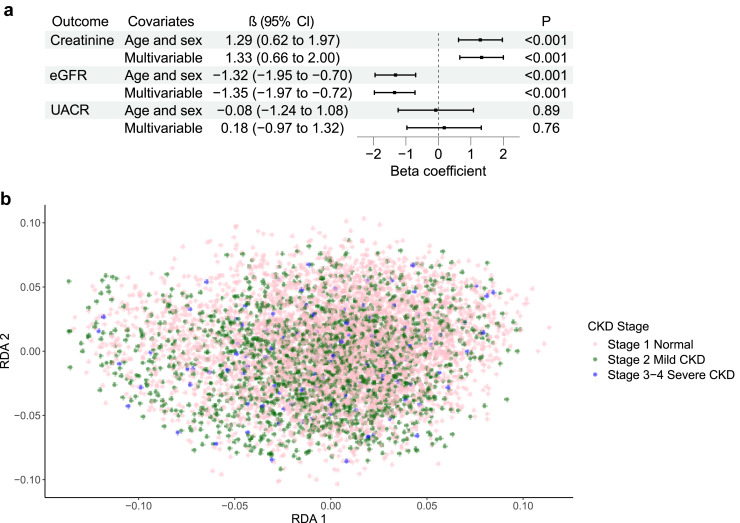
(a) The association of gut microbiome alpha diversity with serum creatinine, eGFR and UACR. (b) The association between beta diversity (redundancy 1 and 2) and CKD stage (stage 1: eGFR > 90 ml/min per 1.73 m^2^; stage 2: eGFR, 60–89 ml/min per 1.73 m^2^; and stage 3–4: eGFR < 60 ml/min per 1.73 m^2^). CKD, chronic kidney disease; dbRDA, redundancy analysis; eGFR, estimated glomerular filtration rate; UACR, urine albumin-to-creatinine ratio.

Gut microbiome alpha diversity was positively associated with the baseline SCR and negatively with eGFR in both the age- and sex-adjusted and the multivariable-adjusted models (Figure 1a). Beta diversity was associated with baseline SCR and eGFR in both the age- and sex-adjusted, and the multivariable-adjusted models (Table 2). Alpha and beta diversity were not associated with UACR (Figure 1 and Table 2).

**Table 2:** The associations of beta diversity with baseline kidney function and incident CKD

Exposure	Age- and sex-adjusted				Multivariable-adjusted			
	*n*	F	*P*-value	R^2^	*n*	F	*P*-value	R^2^
Baseline analyses								
eGFR	6699	3.30	0.001	0.05	6699	3.17	0.001	0.04
Serum creatinine	6699	2.84	0.001	0.04	6699	2.82	0.001	0.04
UACR	797	1.17	0.27	0.14	797	1.00	0.41	0.12
Prospective analyses								
Incident CKD	6556	1.38	0.08	0.02	6556	1.41	0.09	0.02

CKD, chronic kidney disease; eGFR, estimated glomerular filtration rate; UACR, urinary albumin-creatinine ratio.

**Figure 2:**
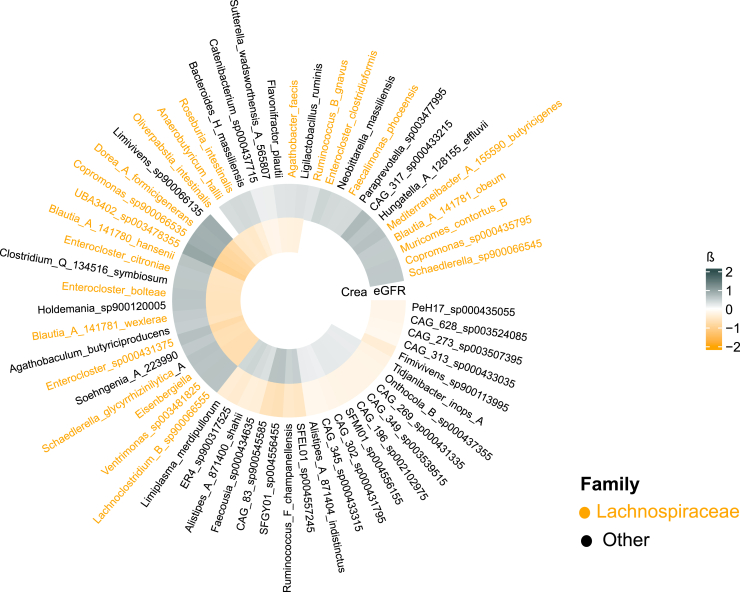
Species associated with baseline serum creatinine (inner circle) and eGFR (outer circle). Crea, creatinine; eGFR, estimated glomerular filtration rate. Gray indicates high abundance and orange indicates low abundance.

In taxa-level analyses for baseline kidney function, 43 significant species-level, 55 genus-level, 19 family-level and 4 phylum-level associations for baseline SCR were observed (false discovery rate: *P* < 0.05 for all; Figure 2 and Supplementary Tables S2–S5). Of the 43 species-level associations, 26 (60.5%) were negative and 17 (39.5%) were positive. Notably, 16 (37.2%) of the 43 species associated with baseline SCR belonged to the *Lachnospiraceae* family and all these associations were negative. Concordantly, the *Lachnospiraceae* family was negatively associated with baseline SCR in the family-level analyses (Supplementary Table S4). The results were mainly similar in terms of kidney function when eGFR was used as the outcome variable (Figure 2). No species, genus, family or phylum-level associations with baseline UACR were observed.

In the functional analyses, the abundance of coenzyme A (CoA) biosynthesis I pathway in the species *Roseburia faecis* was significantly associated with baseline SCR (hazard ratio per 1-SD increment: 0.22; 95% CI: 0.11–0.45; false discovery rate *P* = 0.04) when analyzed as a dichotomous variable (Supplementary Table S6). No functional pathways were associated with baseline UACR (Supplementary Tables S7).

### Prospective Analyses

**Figure 3:**
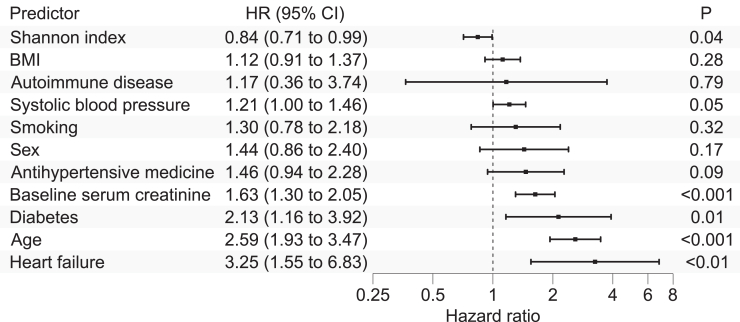
The association of alpha diversity (Shannon index) and conventional CKD risk factors with incident CKD. BMI, body mass index; CI, confidence interval; HR, hazard ratio. Hazard ratios for continuous variables are reported per 1 SD increment.

The baseline characteristics for subjects with and without incident CKD are reported in Table 1. A total of 108 participants developed incident CKD over a median follow-up of 18.6 years. Increased gut microbiome alpha diversity was associated with lower risk of incident CKD in the age- and sex-adjusted model (hazard ratio per 1-SD increment: 0.81; 95% CI: 0.69–0.95; *P* = 0.01) and in the multivariable-adjusted model (hazard ratio per 1-SD increment: 0.84; 95% CI: 0.71–0.99; *P* = 0.04) (Figure 3). No association between gut microbiome beta diversity and incident CKD was observed (Table 2).

In taxa-level Cox survival analyses, we observed no associations of microbial species, genera, families, or phyla with incident CKD. The top 10 species-level associations with incident CKD are summarized in Table 3. No associations between incident CKD and microbial species, genera, families, or phyla were observed in the ANCOM-BC2 analyses for both age- and sex-adjusted and multivariable models. No pathways significantly associated incident CKD were observed in the functional pathway analyses (Supplementary Table S8).

**Table 3:** Top 10 species-level associations with incident CKD in multivariable Cox model

Species	HR	95% CI	FDR-*P*
*Ruminococcus_E_sp003438075*	1.20	1.06–1.36	0.75
*Parabacteroides_B_862066_distasonis*	0.85	0.76–0.96	0.75
*Phascolarctobacterium_A_faecium*	1.10	1.02–1.18	0.75
*CAG_95_sp900066375*	0.66	0.47–0.93	0.75
*CAG_313_sp000433035*	0.91	0.84–0.99	0.75
*Butyrivibrio_A_180067_crossotus*	0.82	0.69–0.97	0.75
*Merdousia_gallistercoris*	1.10	1.01–1.19	0.75
*Alistipes_A_871400_putredinis*	0.89	0.80–0.99	0.75
*Prevotella_lascolaii*	1.19	1.02–1.40	0.75
*UBA738_sp004557735*	0.88	0.78–0.99	0.75

CKD, chronic kidney disease; CI, confidence interval; FDR, false discovery rate; HR, hazard ratio.

## Discussion

This large population-based study investigated the cross-sectional associations between gut microbiome and baseline kidney function and, for the first time, prospective links between gut microbiome and subsequent CKD. As a novel finding, we observed that gut microbiome alpha diversity was negatively associated with incident CKD whereas beta diversity was not. Furthermore, gut microbiome alpha and beta diversity were linked with baseline SCR, but not with baseline UACR. In the cross-sectional analyses, alpha diversity was positively associated with SCR. Numerous taxonomic associations with baseline SCR were observed whereas none were detected for incident CKD. Interestingly, a third of the species that were cross-sectionally negatively associated with baseline SCR belonged to the *Lachnospiraceae* family.

Lower baseline alpha diversity was associated with the risk of developing incident CKD in this largest prospective study to date with a follow-up time > 18 years. This finding is the first of its kind in a prospective population-based study and is intuitive in nature because lower alpha diversity has been linked with prevalent CKD in previous studies.^2^, ^3^, ^4^, ^5^, ^6^ No association between gut microbiome beta diversity and incident CKD was observed in our study. In contrast, in our cross-sectional analyses, lower alpha diversity was associated with better baseline kidney function contrary to most previous works.^2^, ^3^, ^4^, ^5^, ^6^ Our findings therefore serve to highlight the importance of prospective studies in the field of microbiome research for detecting associations and avoiding the biases that involve cross-sectional research settings, such as the nonresponse bias and reverse causation. These biases could explain the mixed results observed in recent studies on the associations between gut microbiome and kidney function.^4^

Limited previous data exist on the association between gut microbiome composition and incident CKD. Experimental animal studies have suggested that changes in the microbiome of the gut may play a role in the development of CKD.^20^ Human studies in this domain are even more sparse and the sample sizes have been small and the follow-up periods short. Recently, Atzeni *et al.*^21^ explored the association between microbiota composition and progression or incidence of CKD during a 1-year follow-up in 343 participants with a mean eGFR of 73 ml/min per 1.73 m^2^. A third of the subjects maintained CKD or proceeded to CKD in the longitudinal analyses. In contrast to our findings, no significant differences in alpha diversity were observed between subjects proceeding to CKD and those who did not.^21^ The reasons behind the discrepant findings on incident CKD between our study and those observed by Atzeni *et al.*^21^ are unclear but likely result from the highly selected study sample in Atzeni *et al.*^21^ Furthermore, our sample size was over 19 times larger and follow-up-period was 18-fold longer compared with the study by Atzeni *et al.*^21^ In addition, the diagnosis of incident CKD was based on a single eGFR measurement after the 1-year follow-up in the study by Atzeni *et al.*^21^; whereas our diagnosis was register-based, which could potentially result in missed diagnoses in both studies. Thus, the next step in prospective gut microbiome research would be to collect serial eGFR measures over a long term for a more granular assessment eGFR decline.^22^ Moreover, the relationship and pathologic processes between gut microbiome and development of CKD require further mechanistic studies.

Gut microbiota of healthy individuals has often been characterized as taxonomically rich, broadly diverse, and stable for the most central taxa.^23^ Microbial alpha diversity has been significantly lower in patients with CKD than in subjects with normal kidney function in most previous studies.^4^ In addition, beta diversity of patients with CKD has been observed to differ from that of healthy subjects in many studies.^4^, ^5^, ^6^ However, the microbiome changes with advancing age, overall disease burden, medications, and diet changes, complicating the interpretation of these cross-sectional microbiome analyses.^24^ In our study, with a randomly selected population sample, greater gut microbiome alpha diversity was associated with higher baseline SCR values. This finding could be partly explained by the fact that eGFR and UACR were within the normal clinical range (≥ 60 ml/min per 1.73 m^2^ and < 3 mg/mmol) in most participants included in our sample. The positive association between alpha diversity and SCR could be a marker for higher baseline muscle mass in our sample compared with patients with CKD. In fact, frailty is a common condition among patients with CKD, and decreased physical, functional, and cognitive reserve along with lower muscle mass, have been associated with decreased gut microbiome diversity.^25^ Higher UACR has been linked to lower microbial diversity in the gut but no associations between alpha diversity and UACR were observed in our study. This finding could partly be due to the smaller sample size and the low number of individuals with an elevated UACR.^6^

We observed a significant association between beta diversity of gut microbiome and baseline kidney function in terms of SCR and eGFR but not with UACR in the cross-sectional analyses. Our findings are in line with a recent systematic review by Zhao *et al.*^4^ covering 25 studies with 918 healthy controls and 1436 patients with CKD, a third of whom had end-stage kidney disease. Altogether, 17 of the 25 studies, 11 comparing patients with CKD and 6 comparing patients with end-stage kidney disease patients with healthy subjects, reported data on gut microbiome beta diversity and in all but 2 a significant difference in beta diversity between the groups was observed.^4^ Our findings reinforce the association between gut microbiome beta diversity and kidney function because the cohort size in our study is many times larger than in previous works with similar findings. Furthermore, the baseline kidney function in our cohort was better than in previous studies, suggesting that the link with beta diversity may exist already at early stages of CKD.

The previously observed taxonomic associations between human gut microbiome and CKD have often been conflicting and difficult to replicate despite considerable research. These discrepancies are at least partly explained by the methodological differences related to sequencing, taxonomic binning, and bioinformatics. At the phylum-level, abundances of *Actinobacteria*, *Firmicutes*, and *Proteobacteria* increased whereas *Bifidobacteria* and *Lactobacilli* decreased in patients with advanced CKD compared with healthy controls in one of the most cited studies examining microbiome changes in advanced CKD.^2^ In line with these findings, we observed negative associations between *Firmicutes* and baseline SCR. However, a more recent systematic review that included 25 studies on microbiome-CKD associations reported an increased abundance in *Proteobacteria* in patients with advanced CKD but inconsistent results in relation to *Firmicutes* compared with controls with normal kidney function.^4^ The progression of kidney function decline appears to affect gut microbiome because the findings on *Firmicutes* and *Actinobacteria* in patients with less severe CKD than in controls were even more inconsistent.^4^ These findings, together with our results, highlight the challenges related to cross-sectional studies in this domain, such as the impact of medications and the disease itself on gut microbiome.

Interestingly, a third of the species-level associations with baseline kidney function observed in our study belonged to the *Lachnospiraceae* family. Although no such association between the *Lachnospiraceae* family and incident CKD was observed in our study, a recent Mendelian randomization study suggested that *Lachnospiraceae* may have protective properties against the development of CKD.^26^ Moreover, the relative abundance of the genera *Lachnoclostridium* and *Lachnospira* were lower in subjects whose kidney function deteriorated in the prospective study by Atzeni *et al.*^21^ In cross-sectional analyses, several human and animal studies have demonstrated a decreased *Lachnospiraceae* abundance in patients with CKD compared with healthy controls.^3^, ^4^, ^5^^,^^20^^,^^26^ Furthermore, lower abundance of *Lachnospiraceae*, and many of the genera and species included in it, has been observed in patients with diabetic kidney disease, kidney stones, IgA nephropathy, membranous nephropathy, or idiopathic nephrosis.^27^, ^28^, ^29^, ^30^ Conversely, patients with CKD or idiopathic nephrosis have demonstrated an increased abundance of genera such as *Lachnospiraceae_ND3007* or *Lachnoclostridium*.^2^^,^^30^^,^^31^ Notably, all species significantly associated with baseline kidney function in our study belonging to the *Lachnospiraceae* family were negatively associated with SCR, that is, better kidney function. This finding may support the hypothesis that some members of the *Lachnospiraceae* family may have kidney-protective effects or thrive better in the presence of desirable kidney function.^26^ Some genera of the *Lachnospiraceae* family, such as the *Blautia*, have been positively associated with trimethylamine-N-oxide, a uremic toxin associated with increased risk of incident CKD.^32^, ^33^, ^34^ Furthermore, several members of the *Lachnospiraceae* family have been positively associated with the risk of type 2 diabetes.^35^ Interestingly, the genus *Lachnospira* of the *Lachnospiraceae* family performed well in differentiating between CKD and healthy controls in a previous study.^36^ All these data appear to suggest that the *Lachnospiraceae* family and some species belonging to it may play a central role in the gut-kidney axis. However, different species belonging to the same family may have opposing associations with kidney outcomes.

Several dietary interventions have been shown to influence *Lachnospiraceae* abundance. Mediterranean diet complemented with ketoanalog supplements and short-term low-phosphorus diet have resulted in an increase in *Lachnospiraceae* abundance, whereas a decrease in abundance has been achieved with a low-protein diet.^37^, ^38^, ^39^ Whether these dietary interventions translate to benefit in kidney outcomes remains unknown because of the multifunctional and conflicting nature of the *Lachnospiraceae* family in relation to kidney outcomes. Interestingly, the use of the sodium glucose cotransporter 2 inhibitor, canagliflozin has resulted in an increase in *Lachnospiraceae UCG-001* abundance in diabetic mice and in improved kidney outcomes in patients with CKD and type 2 diabetes.^40^^,^^41^ Finally, the resolution of kidney function in kidney transplant patients appears to be associated with increased *Lachnospiraceae* abundance, suggesting that these taxa may at least serve as a marker for kidney health.^42^

In the functional analyses, the abundance of prokaryotic biosynthesis I pathway of CoA in the species *Roseburia faecis* was positively associated with baseline SCR. CoA plays a central role in the oxidation of fatty acids as well as that of pyruvate in the citric acid cycle,^43^ and the acetylated form of CoA (acetyl CoA) is essential for many key metabolic processes in living organisms. The decreased abundance of *Roseburia faecis* has been associated with CKD in previous works.^44^ The implications of the link between the biosynthesis of CoA in *Roseburia faecis* and baseline kidney function are difficult to discern, especially because CoA biosynthesis is a primary metabolic process and because the species *Roseburia faecis* was not associated with baseline SCR in our study.

### Limitations

Despite its strengths, our study has some limitations inherent in observational microbiome research. The strengths of our study include a prospective, population-based cohort sample that is the first to-date investigating the links between gut microbiome and baseline kidney function as well as incident CKD. Moreover, the methodology of our study included whole genome, instead of 16S rRNA, sequencing of the fecal samples which allows for a more complete assessment of gut microbiome, including functional and species-level analyses. As a limitation, gut microbiome was analyzed only at a single time point in our study. Furthermore, the diagnoses of incident CKD were based on register data, which could result in missing some less severe cases of incident CKD. However, the quality of the Finnish register data has been shown to be good and captures cases of end-stage CKD with great certainty.^16^ Our analyses on UACR had lower statistical power than those on kidney function because of missing data and largely normal UACRs values. However, such characteristics are typical in population-based samples and our UACR sample is still larger than in most previous studies. We therefore believe these data provide ample opportunities to examine especially the taxonomic relationships between the microbiome of the gut and kidney function in a large, unselected sample of relatively healthy subjects. In line with this reasoning, our prospective and cross-sectional findings are observational and offer new hypotheses for experimental studies and for the research landscape of the human gut microbiome.

## Conclusion

We conclude that alpha diversity of the human gut microbiome was associated with subsequent CKD, suggesting that lower microbiome diversity or factors related to it may have an impact on the development of CKD in the future. Furthermore, alpha and beta diversity of the microbiome and 43 microbial species were associated with baseline SCR. A third of these associations were related to species from the *Lachnospiraceae* family and all were negative, suggesting a possible link between the *Lachnospiraceae* family and desirable kidney health. Our results extend previous cross-sectional studies and help to establish the basis for examining gut microbiome as a CKD risk factor.

## Disclosure

TH has received consulting, lecturing, and authoring fees from Astellas, AstraZeneca, GSK, MSD, Boehringer-Ingelheim, and support for congress attendance from AstraZeneca. RK is a scientific advisory board member, and consultant for BiomeSense, Inc.; has equity and receives income. He is a scientific advisory board member and has equity in GenCirq. He is a consultant for DayTwo and receives income. He has equity in and acts as a consultant for Cybele. He is a cofounder of Biota, Inc., and has equity. He is a cofounder of Micronoma and has equity and is a scientific advisory board member. TN has received consulting and authoring fees from AstraZeneca, Servier Finland, and Orion Corporation. All the other authors declared no competing interests.

## Data Availability Statement

The metagenomic data are available from the European Genome-Phenome Archive (accession number EGAD00001007035). The phenotype data contain sensitive information from health care registers and they are available through the THL biobank upon submission of a research plan and signing a data transfer agreement (https://thl.fi/en/research-and-development/thl-biobank/for-researchers/application-process).

## Funding

L-FY has received a research grant cofunded by the European Union’s Horizon Europe Framework program for research and innovation 2021–2027 under the Marie Skłodowska-Curie grant agreement No 101126611. JP has received research grants from the Paavo Nurmi Foundation and the Finnish Medical Foundation. AH has received grants from the Academy of Finland, the ERC and MSCA Doctoral Network 2023 and is a partner in the “HoloGen” network. KP has received a postdoctoral grant [348439] from the Research Council of Finland. VS has received a research grant from the Juho Vainio Foundation. TN has received funding for this work from the Finnish Research Council, Sigrid Jusélius Foundation, Finnish Foundation for Cardiovascular Research and The Wellbeing Services County of Southwest Finland.

## Supplementary Material

Supplementary file (PDF) **Supplementary Methods.** Further information on the selection of the study sample and methodology regarding the outcome variables**. Supplementary References. Table S1.** Definitions of register-based comorbidities and outcomes. **Table S2.** The significant taxonomic species-level associations between gut microbiome and baseline serum creatinine. **Table S3.** The significant taxonomic genus-level associations between gut microbiome and baseline serum creatinine. **Table S4.** The significant taxonomic family-level associations between gut microbiome and baseline serum creatinine. **Table S5.** The significant taxonomic phylum-level associations between gut microbiome and baseline serum creatinine. **Table S6.** Linear regression model for top 10 functional pathway abundance and baseline serum creatinine using dichotomous variable and inverse-rank normalization. **Table S7.** Linear regression model for top 10 functional pathway abundance and UACR using dichotomous variable and inverse-rank normalization. **Table S8.** Cox proportional hazards ratio model for top 10 functional pathway abundance and incident CKD using dichotomous variable and inverse-rank normalization. STROBE Checklist.

## Footnotes

Supplementary file (PDF)
**Supplementary Methods.** Further information on the selection of the study sample and methodology regarding the outcome variables.
**Supplementary References.**
**Table S1.** Definitions of register-based comorbidities and outcomes. **Table S2.** The significant taxonomic species-level associations between gut microbiome and baseline serum creatinine. **Table S3.** The significant taxonomic genus-level associations between gut microbiome and baseline serum creatinine. **Table S4.** The significant taxonomic family-level associations between gut microbiome and baseline serum creatinine. **Table S5.** The significant taxonomic phylum-level associations between gut microbiome and baseline serum creatinine. **Table S6.** Linear regression model for top 10 functional pathway abundance and baseline serum creatinine using dichotomous variable and inverse-rank normalization. **Table S7.** Linear regression model for top 10 functional pathway abundance and UACR using dichotomous variable and inverse-rank normalization. **Table S8.** Cox proportional hazards ratio model for top 10 functional pathway abundance and incident CKD using dichotomous variable and inverse-rank normalization. STROBE Checklist.

## References

[1] Hill N.R., Fatoba S.T., Oke J.L. (2016). Global prevalence of chronic kidney disease–a systematic review and meta-analysis. PLoS One.

[2] Vaziri N.D., Wong J., Pahl M. (2013). Chronic kidney disease alters intestinal microbial flora. Kidney Int.

[3] Wu I.W., Gao S.S., Chou H.C. (2020). Integrative metagenomic and metabolomic analyses reveal severity-specific signatures of gut microbiota in chronic kidney disease. Theranostics.

[4] Zhao J., Ning X., Liu B., Dong R., Bai M., Sun S. (2021). Specific alterations in gut microbiota in patients with chronic kidney disease: an updated systematic review. Ren Fail.

[5] Ren Z., Fan Y., Li A. (2020). Alterations of the human gut microbiome in chronic kidney disease. Adv Sci (Weinh).

[6] Peters B.A., Qi Q., Usyk M. (2023). Association of the gut microbiome with kidney function and damage in the Hispanic Community Health Study/Study of Latinos (HCHS/SOL). Gut Microbes.

[7] Bartochowski P., Gayrard N., Bornes S. (2022). Gut-kidney axis investigations in animal models of chronic kidney disease. Toxins (Basel).

[8] Forslund S.K., Chakaroun R., Zimmermann-Kogadeeva M. (2021). Combinatorial, additive and dose-dependent drug-microbiome associations. Nature.

[9] Borodulin K., Vartiainen E., Peltonen M. (2015). Forty-year trends in cardiovascular risk factors in Finland. Eur J Public Health.

[10] Salosensaari A., Laitinen V., Havulinna A.S. (2021). Taxonomic signatures of cause-specific mortality risk in human gut microbiome. Nat Commun.

[11] Marotz L., Schwartz T., Thompson L. Earth microbiome project (EMP) high throughput (HTP) DNA extraction protocol. https://repository.oceanbestpractices.org/handle/11329/953.

[12] McDonald D., Jiang Y., Balaban M. (2024). Greengenes2 unifies microbial data in a single reference tree. Nat Biotechnol.

[13] Huang R., Soneson C., Ernst F.G.M. (2021). TreeSummarizedExperiment: a S4 class for data with hierarchical structure. F1000Res.

[14] Caspi R., Billington R., Keseler I.M. (2020). The MetaCyc database of metabolic pathways and enzymes-a 2019 update. Nucleic Acids Res.

[15] Beghini F., McIver L.J., Blanco-Míguez A. (2021). Integrating taxonomic, functional, and strain-level profiling of diverse microbial communities with bioBakery 3. eLife.

[16] Sund R. (2012). Quality of the Finnish Hospital Discharge Register: a systematic review. Scand J Public Health.

[17] R Core Team (2017). R: A Language and Environment for Statistical Computing. R Foundation for Statistical Computing, Vienna. https://www.R-project.org/.

[18] Ernst F., Shetty S., Borman T. (Published 2024). mia: microbiome analysis. R Package Version 1.13.31. GitHub.

[19] Lin H., Peddada S.D. (2024). Multigroup analysis of compositions of microbiomes with covariate adjustments and repeated measures. Nat Methods.

[20] Randall D.W., Kieswich J., Hoyles L., McCafferty K., Curtis M., Yaqoob M.M. (2023). Gut dysbiosis in experimental kidney disease: a meta-analysis of rodent repository data. J Am Soc Nephrol.

[21] Atzeni A., Díaz-López A., Cacho A.H. (2024). Gut microbiota dynamics and association with chronic kidney disease: a longitudinal study within the PREDIMED-Plus trial. Life Sci.

[22] Sundström J., Bodegard J., Bollmann A. (2022). CaReMe CKD investigators. Prevalence, outcomes, and cost of chronic kidney disease in a contemporary population of 2.4 million patients from 11 countries: the CaReMe CKD study. Lancet Reg Health Eur.

[23] Fan Y., Pedersen O. (2021). Gut microbiota in human metabolic health and disease. Nat Rev Microbiol.

[24] Hou K., Wu Z.X., Chen X.Y. (2022). Microbiota in health and diseases. Signal Transduct Target Ther.

[25] Jackson M.A., Jeffery I.B., Beaumont M. (2016). Signatures of early frailty in the gut microbiota. Genome Med.

[26] Ren F., Jin Q., Jin Q. (2023). Genetic evidence supporting the causal role of gut microbiota in chronic kidney disease and chronic systemic inflammation in CKD: a bilateral two-sample Mendelian randomization study. Front Immunol.

[27] Wang Y., Zhao J., Qin Y. (2022). The specific alteration of gut microbiota in diabetic kidney diseases-a systematic review and meta-analysis. Front Immunol.

[28] Stanford J., Charlton K., Stefoska-Needham A., Ibrahim R., Lambert K. (2020). The gut microbiota profile of adults with kidney disease and kidney stones: a systematic review of the literature. BMC Nephrol.

[29] Dong R., Bai M., Zhao J., Wang D., Ning X., Sun S. (2020). A comparative study of the gut microbiota associated with immunoglobulin A nephropathy and membranous nephropathy. Front Cell Infect Microbiol.

[30] He H., Lin M., You L. (2021). Gut microbiota profile in adult patients with idiopathic nephrotic syndrome. BioMed Res Int.

[31] Pourafshar S., Sharma B., Allen J. (2024). Longitudinal pilot evaluation of the gut microbiota comparing patients with and without chronic kidney disease. J Ren Nutr.

[32] Vacca M., Celano G., Calabrese F.M., Portincasa P., Gobbetti M., De Angelis M. (2020). The controversial role of human gut Lachnospiraceae. Microorganisms.

[33] Fu B.C., Hullar M.A.J., Randolph T.W. (2020). Associations of serum trimethylamine N-oxide, choline, carnitine, and betaine with inflammatory and cardiometabolic risk biomarkers and the fecal microbiome in the Multiethnic Cohort Adiposity Phenotype Study. Am J Clin Nutr.

[34] Wang M., Tang W.H.W., Li X.S. (2024). The gut microbial metabolite trimethylamine N -oxide, incident CKD, and kidney function decline. J Am Soc Nephrol.

[35] Ruuskanen M.O., Erawijantari P.P., Havulinna A.S. (2022). Gut microbiome composition is predictive of incident type 2 diabetes in a population cohort of 5,572 Finnish adults. Diabetes Care.

[36] Lun H., Yang W., Zhao S. (2019). Altered gut microbiota and microbial biomarkers associated with chronic kidney disease. MicrobiologyOpen.

[37] Rocchetti M.T., Di Iorio B.R., Vacca M. (2021). Ketoanalogs’ effects on intestinal microbiota modulation and uremic toxins serum levels in chronic kidney disease (Medika2 study). J Clin Med.

[38] Zhang J.Y., Niu C., Zhang Q. (2021). Full-scale clinical data and reshaped intestinal microbiome on a short-term low-phosphorus diet among healthy adults. J Ren Nutr.

[39] Wu I.W., Lee C.C., Hsu H.J. (2020). Compositional and functional adaptations of intestinal microbiota and related metabolites in CKD patients receiving dietary protein restriction. Nutrients.

[40] Zeng L., Ma J., Wei T. (2024). The effect of canagliflozin on gut microbiota and metabolites in type 2 diabetic mice. Genes Genomics.

[41] Perkovic V., Jardine M.J., Neal B. (2019). Canagliflozin and renal outcomes in type 2 diabetes and nephropathy. N Engl J Med.

[42] Sivaraj S., Chan A., Pasini E. (2020). Enteric dysbiosis in liver and kidney transplant recipients: a systematic review. Transpl Int.

[43] Barritt S.A., DuBois-Coyne S.E., Dibble C.C. (2024). Coenzyme A biosynthesis: mechanisms of regulation, function and disease. Nat Metab.

[44] Lohia S., Vlahou A., Zoidakis J. (2022). Microbiome in chronic kidney disease (CKD): an omics perspective. Toxins (Basel).

