# Supplementary material for: Gut Microbiome as a Risk Factor for Future CKD
Source: Kidney Int Rep. 2025 Apr 7;10(6):1673–82. doi: 10.1016/j.ekir.2025.04.007 (PMC12231020; doi:10.1016/j.ekir.2025.04.007)
Supplement: Supplementary file (PDF) — Supplementary Methods. Further information on the selection of the study sample and methodology regarding the outcome variables. Supplementary References. Table S1. Definitions of register-based comorbidities and outcomes. Table S2. The significant taxonomic species-level associations between gut microbiome and baseline serum creatinine. Table S3. The significant taxonomic genus-level associations between gut microbiome and baseline serum creatinine. Table S4. The significant taxonomic family-level associations between gut microbiome and baseline serum creatinine. Table S5. The significant taxonomic phylum-level associations between gut microbiome and baseline serum creatinine. Table S6. Linear regression model for top 10 functional pathway abundance and baseline serum creatinine using dichotomous variable and inverse-rank normalization. Table S7. Linear regression model for top 10 functional pathway abundance and UACR using dichotomous variable and inverse-rank normalization. Table S8. Cox proportional hazards ratio model for top 10 functional pathway abundance and incident CKD using dichotomous variable and inverse-rank normalization. STROBE Checklist. [file mmc1.pdf]

Table of Contents

Supplemental Methods. Further information on the selection of the study sample and methodology regarding the outcome variables – Page 2

Supplemental Table S1. Definitions of register-based comorbidities and outcomes - Page 3

Supplementary Table S2. The significant taxonomic species-level associations between gut microbiome and baseline serum creatinine - Pages 4–5

Supplementary Table S3. The significant taxonomic genus-level associations between gut microbiome and baseline serum creatinine - Pages 6–7

Supplemental Table S4. The significant taxonomic family-level associations between gut microbiome and baseline serum creatinine - Pages 8

Supplemental Table S5. The significant taxonomic phylum-level associations between gut microbiome and baseline serum creatinine - Pages 9

Supplementary Table S6. Linear regression model for top 10 functional pathway abundance and baseline serum creatinine using dichotomous variable and inverse-rank normalization - Pages 10–11

Supplementary Table S7. Linear regression model for top 10 functional pathway abundance and UACR using dichotomous variable and inverse-rank normalization - Pages 12–13

Supplementary Table S8. Cox proportional hazards ratio model for top 10 functional pathway abundance and incident CKD using dichotomous variable and inverse-rank normalization - Pages 14–15

Supplementary References Page 16

Supplemental Methods

Study sample

Of the 8,799 study participants, 7054 (80.2%) provided stool samples for microbiome analysis. For the cross-sectional analyses (sample 1), 40 pregnant women, 241 participants who received antibiotics during 1 month preceding the sampling, 62 participants due to incomplete baseline covariate data and 12 due to low (N < 35,000) total read count were excluded, resulting in a sample of 6699 individuals. In the cross-sectional analyses for urine albumin-creatinine (UACR), a sample of 797 individuals (sample 2) was included, as the rest were missing urine samples. In the prospective analyses performed in participants without prior diagnosis of CKD, an additional 5 participants with prevalent register-based CKD, 111 participants with an estimated glomerular filtration rate (eGFR) < 60 ml/min/1.73m^2, and 27 participants with UACR ≥ 3 mg/mmol were excluded from sample 1 for a final sample of 6556 individuals (sample 3).

Outcome variables

Serum samples for creatinine were collected from all participants and stored frozen at -70C until enzymatic analysis in 2007-2008 (Thermo Scientific, Vantaa, Finland) using an Abbott Architect c8000 analyzer. UACR was measured using Cobas Mira analyzer with Albumin/microalbuminuria (Konelab, Espoo, Finland) and ABX Pentra Creatinine 80 (Horiba, Kyoto, Japan) as reagents. eGFR was not considered a primary outcome measure as all analyses were adjusted for age and gender, the other two components besides serum creatinine in the contemporary Chronic Kidney Disease Epidemiology Collaboration (CKD-EPI) algorithm for eGFR (S1).

Supplemental Table S1. Definitions of register-based comorbidities and outcomes.

Comorbidity/Outcome	Hospital discharge and death registers	Drug reimbursement and purchase registers
Diabetes	ICD-10: E10-E14<br>ICD-9: 250<br>ICD-8: 250	ATC: A10, A10A, A10B<br>DR: 103 (hypertension)
Heart failure	ICD-10: I50, I110, I130, I132<br>ICD-9: 4029B, 4148, 428<br>ICD-8: 42700, 42710, 428	ATC: C03CA01, C03EB01;<br>DR: 201 (heart failure)
Autoimmune disease	ICD-10: M32, K50, K51, M05, M06, M351<br>ICD-9: 7100A, 555, 556, 7140A, 7140B, 7241, 7142<br>ICD-8: 734, 5630, 5631, 7121, 7122, 7123	DR: 208 (ulcerative colitis and Crohn’s disease)
CKD	ICD-10: N18, N19<br>ICD-9: 585<br>ICD-8: 58200<br>NCSP: PBL (arteriovenous fistula creation), JAK10 (peritoneal dialysis catheter insertion), PG5RT (arteriovenous fistula percutaneous transluminal angioplasty), PG5ST (arteriovenous fistula thrombolysis), TJA33 (laparoscopic insertion of a peritoneal dialysis catheter), TJA35 (removal of peritoneal dialysis catheter), TK820 (peritoneal dialysis)	DR: 190, 320 (sevelamer, lanthanum carbonate, sucroferric oxyhydroxide), 137 (medications associated with dialysis therapy), 138 (medications targeting renal anemia), 3066 (Roxadustat), 3071 (finerenone)

Diseases are defined by ICD-codes, ATC-codes, Nordic Classification of Surgical Procedures and Finnish Social Insurance Institution drug codes. ICD, International Classification of Diseases; NCSP, Nordic Classification of Surgical Procedures; ATC, Anatomical therapeutic chemical code; DR, Drug reimbursement code.

Supplementary Table S2. The significant taxonomic species-level associations between gut microbiome and baseline serum creatinine.

Species	Estimate	SE	T-statistic	P-value	95% CI low	95% CI high	FDR P-value
CAG_83_sp900545585	0.644	0.106	6.104	<0.001	0.437	0.851	<0.001
Enterocloster_sp000431375	-0.772	0.137	-5.639	<0.001	-1.040	-0.503	<0.001
ER4_sp900317525	0.456	0.097	4.702	<0.001	0.266	0.647	<0.001
SFEL01_sp004557245	0.511	0.109	4.687	<0.001	0.297	0.724	<0.001
Copromonas_sp900066535	-0.922	0.219	-4.204	<0.001	-1.351	-0.492	0.001
Blautia_A_141781_wexlerae	-0.609	0.147	-4.155	<0.001	-0.896	-0.322	0.001
Ruminococcus_F_champanellensis	0.479	0.115	4.153	<0.001	0.253	0.706	0.001
CAG_196_sp002102975	0.229	0.058	3.949	<0.001	0.115	0.342	0.003
Roseburia_intestinalis	-0.482	0.123	-3.922	<0.001	-0.722	-0.241	0.003
Limiplasma_merdipullorum	0.427	0.117	3.660	<0.001	0.198	0.655	0.006
Dorea_A_formicigenerans	-0.865	0.237	-3.655	<0.001	-1.328	-0.401	0.006
UBA3402_sp003478355	-1.000	0.276	-3.624	<0.001	-1.541	-0.459	0.006
GUT_Genus:Eisenbergiella	-0.693	0.196	-3.530	<0.001	-1.078	-0.308	0.008
Enterocloster_bolteae	-0.592	0.169	-3.512	<0.001	-0.922	-0.262	0.008
Tidjanibacter_inops_A	0.210	0.061	3.462	0.001	0.091	0.329	0.009
Agathobaculum_butyriciproducens	-0.590	0.171	-3.456	0.001	-0.924	-0.255	0.009
SFMI01_sp004556155	0.231	0.067	3.444	0.001	0.100	0.363	0.009
Holdemania_sp900120005	-0.613	0.178	-3.435	0.001	-0.963	-0.263	0.009
CAG_349_sp003539515	0.224	0.069	3.265	0.001	0.089	0.358	0.014
GUT_Genus:Soehngenia_A_223990	-0.678	0.208	-3.257	0.001	-1.086	-0.270	0.014
Alistipes_A_871400_shahii	0.380	0.117	3.253	0.001	0.151	0.609	0.014
Sutterella_wadsworthensis_A_565807	-0.186	0.057	-3.236	0.001	-0.298	-0.073	0.015
Anaerobutyricum_hallii	-0.458	0.150	-3.057	0.002	-0.752	-0.164	0.025
Onthocola_B_sp000437355	0.198	0.065	3.040	0.002	0.070	0.326	0.025
Flavonifractor_plautii	-0.274	0.090	-3.034	0.002	-0.451	-0.097	0.025

Faecousia_sp000434635	0.326	0.108	3.013	0.003	0.114	0.538	0.026
SFGY01_sp004556455	0.653	0.217	3.007	0.003	0.227	1.078	0.026
Catenibacterium_sp000437715	-0.194	0.065	-2.994	0.003	-0.320	-0.067	0.026
Ligilactobacillus_ruminis	-0.303	0.102	-2.964	0.003	-0.504	-0.103	0.028
Enterocloster_citroniae	-0.567	0.194	-2.915	0.004	-0.948	-0.186	0.031
Schaedlerella_glycyrrhizinilytica_A	-0.658	0.227	-2.897	0.004	-1.103	-0.213	0.031
Lachnoclostridium_B_sp900066555	-0.697	0.241	-2.891	0.004	-1.170	-0.224	0.031
Blautia_A_141780_hansenii	-0.560	0.194	-2.884	0.004	-0.941	-0.180	0.031
Clostridium_Q_134516_symbiosum	-0.587	0.204	-2.881	0.004	-0.987	-0.188	0.031
CAG_269_sp000431335	0.220	0.077	2.845	0.004	0.068	0.372	0.033
CAG_345_sp000433315	0.180	0.064	2.820	0.005	0.055	0.304	0.035
Alistipes_A_871404_indistinctus	0.184	0.066	2.808	0.005	0.055	0.312	0.035
Limivivens_sp900066135	-0.774	0.277	-2.793	0.005	-1.317	-0.231	0.036
Bacteroides_H_massiliensis	-0.362	0.130	-2.790	0.005	-0.617	-0.108	0.036
Agathobacter_faecis	-0.283	0.102	-2.768	0.006	-0.483	-0.082	0.037
CAG_302_sp000431795	0.155	0.057	2.728	0.006	0.044	0.267	0.041
Oliverpabstia_intestinalis	-0.483	0.178	-2.717	0.007	-0.832	-0.135	0.041
Ventrimonas_sp003481825	-0.697	0.257	-2.711	0.007	-1.201	-0.193	0.041

Supplementary Table S3. The significant taxonomic genus-level associations between gut microbiome and baseline serum creatinine.

**Genus**	**Estimate**	**SE**	**T-statistic**	**P-value**	**95% CI low**	**95% CI high**	**FDR P-value**
Enterocloster	-0.907	0.162	-5.604	<0.001	-1.224	-0.590	<0.001
CAG_83	0.588	0.120	4.920	<0.001	0.354	0.822	<0.001
SFEL01	0.525	0.113	4.655	<0.001	0.304	0.746	<0.001
Copromonas	-1.060	0.233	-4.551	<0.001	-1.517	-0.604	<0.001
Methanobrevibacter_A	0.287	0.063	4.521	<0.001	0.162	0.411	<0.001
UBA3402	-1.179	0.282	-4.188	<0.001	-1.731	-0.627	0.001
Ruminococcus_F	0.487	0.119	4.086	<0.001	0.253	0.721	0.001
Agathobaculum	-0.856	0.214	-4.001	<0.001	-1.276	-0.437	0.001
Roseburia	-0.614	0.154	-3.991	<0.001	-0.915	-0.312	0.001
Blautia_A_141781	-0.738	0.189	-3.897	<0.001	-1.110	-0.367	0.002
CAG_196	0.225	0.058	3.870	<0.001	0.111	0.339	0.002
Eisenbergiella	-0.743	0.201	-3.690	<0.001	-1.138	-0.348	0.003
Soehngenia_A_223990	-0.759	0.209	-3.633	<0.001	-1.169	-0.350	0.004
Limiplasma	0.425	0.120	3.550	<0.001	0.190	0.659	0.005
Holdemania	-0.653	0.186	-3.515	<0.001	-1.017	-0.289	0.005
SFMI01	0.255	0.073	3.502	<0.001	0.112	0.397	0.005
Limivivens	-1.021	0.294	-3.473	0.001	-1.597	-0.445	0.005
Ligilactobacillus	-0.357	0.105	-3.409	0.001	-0.563	-0.152	0.006
Tidjanibacter	0.205	0.061	3.368	0.001	0.086	0.325	0.007
Ventrimonas	-0.895	0.268	-3.343	0.001	-1.419	-0.370	0.007
Anaerobutyricum	-0.536	0.163	-3.293	0.001	-0.856	-0.217	0.008
Lachnoclostridium_B	-0.835	0.256	-3.263	0.001	-1.337	-0.333	0.009
Clostridium_Q_134516	-0.668	0.205	-3.255	0.001	-1.070	-0.266	0.009
Mediterraneibacter_A_155590	-0.875	0.273	-3.200	0.001	-1.411	-0.339	0.010
CAG_349	0.223	0.070	3.199	0.001	0.086	0.360	0.010
Flavonifractor	-0.277	0.088	-3.127	0.002	-0.450	-0.103	0.012

Dorea_A	-0.590	0.190	-3.097	0.002	-0.963	-0.217	0.012
CAG_313	0.173	0.056	3.080	0.002	0.063	0.283	0.013
Oliverpabstia	-0.555	0.181	-3.073	0.002	-0.910	-0.201	0.013
Longicatena	-0.625	0.206	-3.038	0.002	-1.028	-0.222	0.014
CAG_317_146760	-0.790	0.262	-3.021	0.003	-1.303	-0.278	0.014
14_2	-0.791	0.264	-2.999	0.003	-1.309	-0.274	0.015
Onthocola_B	0.198	0.066	2.980	0.003	0.068	0.328	0.015
CAG_603	-0.697	0.234	-2.976	0.003	-1.156	-0.238	0.015
Schaedlerella	-0.734	0.248	-2.960	0.003	-1.220	-0.248	0.015
Faecousia	0.324	0.111	2.911	0.004	0.106	0.543	0.017
Lawsonibacter	-0.738	0.257	-2.868	0.004	-1.242	-0.234	0.019
CAG_95	-0.788	0.275	-2.861	0.004	-1.328	-0.248	0.019
Hungatella_A_128155	-0.540	0.189	-2.856	0.004	-0.911	-0.169	0.019
Muricomes_149725	-0.650	0.228	-2.849	0.004	-1.096	-0.203	0.019
SFGY01	0.633	0.229	2.771	0.006	0.185	1.081	0.023
Ruminococcus_B	-0.283	0.103	-2.742	0.006	-0.486	-0.081	0.025
CAG_269	0.214	0.078	2.738	0.006	0.061	0.367	0.025
CAG_345	0.175	0.064	2.732	0.006	0.050	0.301	0.025
Holdemanella	-0.316	0.120	-2.642	0.008	-0.551	-0.082	0.032
CAG_302	0.152	0.058	2.628	0.009	0.039	0.266	0.032
Bariatricus	-0.500	0.191	-2.610	0.009	-0.875	-0.124	0.033
Fusicatenibacter	-0.444	0.171	-2.594	0.010	-0.779	-0.108	0.034
CAG_274	-0.156	0.060	-2.581	0.010	-0.274	-0.038	0.035
Blautia_A_141780	-0.542	0.212	-2.558	0.011	-0.957	-0.127	0.036
NSJ_61	-0.858	0.344	-2.492	0.013	-1.533	-0.183	0.043
Neobittarella	-0.367	0.150	-2.447	0.014	-0.662	-0.073	0.047
Agathobacter_164117	-0.302	0.123	-2.444	0.015	-0.543	-0.060	0.047
Catenibacterium	-0.178	0.073	-2.436	0.015	-0.322	-0.035	0.047
Akkermansia	0.186	0.076	2.435	0.015	0.036	0.336	0.047

Supplementary Table S4. The significant taxonomic family-level associations between gut microbiome and baseline serum creatinine.

**Family**	**Estimate**	**SE**	**T-statistic**	**P-value**	**95% CI low**	**95% CI high**	**FDR P-value**
CAG_74	0.530	0.115	4.606	<0.001	0.304	0.755	<0.001
Selenomonadaceae_42771	-0.469	0.102	-4.593	<0.001	-0.669	-0.269	<0.001
Methanobacteriaceae	0.287	0.066	4.385	<0.001	0.159	0.416	<0.001
Lachnospiraceae	-0.797	0.203	-3.921	<0.001	-1.196	-0.399	0.001
Anaerovoracaceae	-1.017	0.264	-3.855	<0.001	-1.534	-0.500	0.001
Megasphaeraceae	-0.466	0.122	-3.825	<0.001	-0.705	-0.227	0.001
Erysipelotrichaceae	-0.812	0.219	-3.704	<0.001	-1.242	-0.382	0.002
Tissierellaceae	-0.643	0.177	-3.627	<0.001	-0.991	-0.296	0.002
Bacteroidaceae	-0.489	0.147	-3.337	0.001	-0.776	-0.202	0.005
UBA660	0.244	0.076	3.223	0.001	0.096	0.392	0.007
CAG_917	0.232	0.073	3.186	0.001	0.089	0.375	0.007
CAG_138	0.433	0.138	3.133	0.002	0.162	0.705	0.008
CAG_313	0.175	0.058	3.014	0.003	0.061	0.290	0.010
Butyricicoccaceae	-0.676	0.226	-2.996	0.003	-1.119	-0.234	0.010
CAG_274	-0.166	0.060	-2.759	0.006	-0.284	-0.048	0.020
Lactobacillaceae	-0.278	0.102	-2.729	0.006	-0.478	-0.078	0.021
CAG_508	0.260	0.099	2.612	0.009	0.065	0.455	0.028
Ruminococcaceae	-0.576	0.223	-2.579	0.010	-1.013	-0.138	0.029
Coprobacillaceae	-0.336	0.139	-2.421	0.016	-0.607	-0.064	0.042

Supplementary Table S5. The significant taxonomic phyla-level associations between gut microbiome and baseline serum creatinine.

Phylum	Estimate	SE	T-statistic	P-value	95% CI low	95% CI high	FDR P-value
Methanobacteriota_A_1229	0.297	0.067	4.446	<0.001	0.166	0.429	<0.001
Firmicutes_C	-0.397	0.118	-3.365	0.001	-0.629	-0.166	0.004
Firmicutes_A	-0.743	0.225	-3.299	0.001	-1.185	-0.302	0.004
Bacteroidota	-0.394	0.165	-2.389	0.017	-0.718	-0.071	0.047

Supplementary Table S6. Linear regression model for top 10 functional pathway abundance and baseline serum creatinine using dichotomous variable and inverse-rank normalization.

Pathway	HR	95% CI	P	FDR-P
Dichotomous variable				
COA-PWY: coenzyme A biosynthesis I (prokaryotic)|g__Roseburia.s__Roseburia_faecis	0.218	0.107 — 0.445	<0.001	0.044
COA-PWY-1: superpathway of coenzyme A biosynthesis III (mammals)|g__Roseburia.s__Roseburia_faecis	0.261	0.124 — 0.548	<0.001	0.143
BRANCHED-CHAIN-AA-SYN-PWY: superpathway of branched chain amino acid biosynthesis|g__Butyrivibrio.s__Butyrivibrio_crossotus	7.095	2.354 — 21.379	0.001	0.143
ILEUSYN-PWY: L-isoleucine biosynthesis I (from threonine)|g__Butyrivibrio.s__Butyrivibrio_crossotus	7.095	2.354 — 21.379	0.001	0.143
PWY-5103: L-isoleucine biosynthesis III|g__Butyrivibrio.s__Butyrivibrio_crossotus	7.095	2.354 — 21.379	0.001	0.143
UNINTEGRATED|g__Roseburia.s__Roseburia_intestinalis	0.308	0.155 — 0.614	0.001	0.143
1CMET2-PWY: folate transformations III (E. coli)|g__Bacteroides.s__Bacteroides_dorei	0.309	0.155 — 0.616	0.001	0.143
PANTOSYN-PWY: superpathway of coenzyme A biosynthesis I (bacteria)|g__Bacteroides.s__Bacteroides_dorei	0.27	0.124 — 0.586	0.001	0.143
DTDPRHAMSYN-PWY: dTDP-β-L-rhamnose biosynthesis|g__Butyrivibrio.s__Butyrivibrio_crossotus	5.989	2.039 — 17.588	0.001	0.143
PWY-6123: inosine-5'-phosphate biosynthesis I|g__Bacteroides.s__Bacteroides_dorei	0.326	0.165 — 0.643	0.001	0.143
Inverse rank normalization				
COA-PWY: coenzyme A biosynthesis I (prokaryotic)|g__Roseburia.s__Roseburia_faecis	0.444	0.294 — 0.67	<0.001	0.167
COA-PWY-1: superpathway of coenzyme A biosynthesis III (mammals)|g__Roseburia.s__Roseburia_faecis	0.484	0.318 — 0.738	0.001	0.183
UNINTEGRATED|g__Roseburia.s__Roseburia_intestinalis	0.509	0.34 — 0.761	0.001	0.183
BRANCHED-CHAIN-AA-SYN-PWY: superpathway of branched chain amino acid biosynthesis|g__Butyrivibrio.s__Butyrivibrio_crossotus	2.468	1.435 — 4.246	0.001	0.183

PWY-5103: L-isoleucine biosynthesis III|g__Butyrivibrio.s__Butyrivibrio_crossotus	2.467	1.434 — 4.244	0.001	0.183
PWY-5100: pyruvate fermentation to acetate and lactate II|g__Roseburia.s__Roseburia_faecis	0.488	0.318 — 0.751	0.001	0.183
ILEUSYN-PWY: L-isoleucine biosynthesis I (from threonine)|g__Butyrivibrio.s__Butyrivibrio_crossotus	2.461	1.431 — 4.233	0.001	0.183
PWY-1042: glycolysis IV|g__Roseburia.s__Roseburia_faecis	0.509	0.332 — 0.782	0.002	0.183
ASPASN-PWY: superpathway of L-aspartate and L-asparagine biosynthesis|g__Bacteroides.s__Bacteroides_dorei	0.563	0.389 — 0.813	0.002	0.183
DTDPRHAMSYN-PWY: dTDP-β-L-rhamnose biosynthesis|g__Butyrivibrio.s__Butyrivibrio_crossotus	2.296	1.346 — 3.918	0.002	0.183

Supplementary Table S7. Linear regression model for top 10 functional pathway abundance and UACR using dichotomous variable and inverse-rank normalization.

Pathway	HR	95% CI	P	FDR-P
Dichotomous variable				
PWY-7539: 6-hydroxymethyl-dihydropterin diphosphate biosynthesis III (Chlamydia)|g__Alistipes.s__Alistipes_putredinis	0.718	0.599 — 0.859	<0.001	0.479
PWY-7221: guanosine ribonucleotides de novo biosynthesis|g__Blautia.s__Ruminococcus_torques	0.838	0.746 — 0.941	0.003	0.568
RHAMCAT-PWY: L-rhamnose degradation I|g__Bacteroides.s__Bacteroides_thetaiotaomicron	1.32	1.096 — 1.591	0.004	0.568
PWY-6147: 6-hydroxymethyl-dihydropterin diphosphate biosynthesis I|g__Alistipes.s__Alistipes_putredinis	0.813	0.706 — 0.936	0.004	0.568
PANTO-PWY: phosphopantothenate biosynthesis I|g__Blautia.s__Ruminococcus_torques	0.833	0.735 — 0.944	0.004	0.568
PEPTIDOGLYCANSYN-PWY: peptidoglycan biosynthesis I (meso-diaminopimelate containing)|g__Alistipes.s__Alistipes_putredinis	0.827	0.726 — 0.942	0.004	0.568
GLUTORN-PWY: L-ornithine biosynthesis I|g__Blautia.s__Ruminococcus_torques	0.838	0.742 — 0.947	0.005	0.568
RIBOSYN2-PWY: flavin biosynthesis I (bacteria and plants)|g__Blautia.s__Ruminococcus_torques	0.84	0.745 — 0.948	0.005	0.568
PANTOSYN-PWY: superpathway of coenzyme A biosynthesis I (bacteria)|g__Alistipes.s__Alistipes_putredinis	0.777	0.653 — 0.925	0.005	0.568
NONMEVIPP-PWY: methylerythritol phosphate pathway I|g__Alistipes.s__Alistipes_putredinis	0.797	0.681 — 0.934	0.005	0.568
Inverse rank normalization				
PWY-7539: 6-hydroxymethyl-dihydropterin diphosphate biosynthesis III (Chlamydia)|g__Alistipes.s__Alistipes_putredinis	0.839	0.764 — 0.921	<0.001	0.281
RHAMCAT-PWY: L-rhamnose degradation I|g__Bacteroides.s__Bacteroides_thetaiotaomicron	1.169	1.068 — 1.28	0.001	0.281
PEPTIDOGLYCANSYN-PWY: peptidoglycan biosynthesis I (meso-diaminopimelate containing)|g__Alistipes.s__Alistipes_putredinis	0.879	0.815 — 0.948	0.001	0.281
HISDEG-PWY: L-histidine degradation I|g__Alistipes.s__Alistipes_putredinis	0.889	0.829 — 0.954	0.001	0.281

PWY-5030: L-histidine degradation III|g__Alistipes.s__Alistipes_putredinis	0.884	0.821 — 0.953	0.001	0.281
NONMEVIPP-PWY: methylerythritol phosphate pathway I|g__Alistipes.s__Alistipes_putredinis	0.869	0.798 — 0.947	0.001	0.281
PANTOSYN-PWY: superpathway of coenzyme A biosynthesis I (bacteria)|g__Alistipes.s__Alistipes_putredinis	0.862	0.787 — 0.945	0.002	0.281
PWY-6147: 6-hydroxymethyl-dihydropterin diphosphate biosynthesis I|g__Alistipes.s__Alistipes_putredinis	0.878	0.81 — 0.951	0.002	0.281
PWY-7221: guanosine ribonucleotides de novo biosynthesis|g__Blautia.s__Ruminococcus_torques	0.891	0.829 — 0.958	0.002	0.281
PWY-6386: UDP-N-acetylmuramoyl-pentapeptide biosynthesis II (lysine-containing)|g__Alistipes.s__Alistipes_putredinis	0.882	0.815 — 0.956	0.002	0.281

Supplementary Table S8. Cox proportional hazards ratio model for top 10 functional pathway abundance and incident CKD using dichotomous variable and inverse-rank normalization.

Pathway	HR	95% CI	P	FDR-P
Dichotomous variable				
PWY-6527: stachyose degradation|g__Lachnospiraceae_unclassified.s__Eubacterium_rectale	2.309	1.476 — 3.612	<0.001	0.191
PWY-6527: stachyose degradation|g__Blautia.s__Ruminococcus_torques	2.099	1.268 — 3.475	0.004	0.895
PWY-7220: adenosine deoxyribonucleotides de novo biosynthesis II|g__Bacteroides.s__Bacteroides_caccae	0.348	0.152 — 0.797	0.012	0.895
PWY-7222: guanosine deoxyribonucleotides de novo biosynthesis II|g__Bacteroides.s__Bacteroides_caccae	0.348	0.152 — 0.797	0.012	0.895
PWY-7228: superpathway of guanosine nucleotides de novo biosynthesis I|g__Bacteroides.s__Bacteroides_caccae	0.365	0.16 — 0.836	0.017	0.895
PWY-7221: guanosine ribonucleotides de novo biosynthesis|g__Bacteroides.s__Bacteroides_vulgatus	0.631	0.425 — 0.936	0.022	0.895
PWY-5973: cis-vaccenate biosynthesis|g__Bacteroides.s__Bacteroides_stercoris	0.311	0.114 — 0.848	0.022	0.895
PWY-6124: inosine-5'-phosphate biosynthesis II|g__Bacteroides.s__Bacteroides_stercoris	0.351	0.143 — 0.865	0.023	0.895
PANTO-PWY: phosphopantothenate biosynthesis I|g__Bacteroides.s__Bacteroides_caccae	0.263	0.083 — 0.833	0.023	0.895
PWY-6124: inosine-5'-phosphate biosynthesis II|g__Bacteroides.s__Bacteroides_ovatus	0.508	0.282 — 0.915	0.024	0.895
Inverse rank normalization				
PWY-6527: stachyose degradation|g__Lachnospiraceae_unclassified.s__Eubacterium_rectale	1.531	1.222 — 1.919	0	0.168
PWY-6527: stachyose degradation|g__Blautia.s__Ruminococcus_torques	1.41	1.089 — 1.828	0.009	0.994
UNINTEGRATED|g__Butyrivibrio.s__Butyrivibrio_crossotus	0.533	0.313 — 0.91	0.021	0.994
PWY-7220: adenosine deoxyribonucleotides de novo biosynthesis II|g__Bacteroides.s__Bacteroides_caccae	0.585	0.37 — 0.925	0.022	0.994

PWY-7222: guanosine deoxyribonucleotides de novo biosynthesis II|g__Bacteroides.s__Bacteroides_caccae	0.585	0.37 — 0.925	0.022	0.994
PWY-5973: cis-vaccenate biosynthesis|g__Bacteroides.s__Bacteroides_stercoris	0.517	0.291 — 0.92	0.025	0.994
PWY-6124: inosine-5'-phosphate biosynthesis II|g__Bacteroides.s__Bacteroides_stercoris	0.547	0.323 — 0.926	0.025	0.994
PWY-7228: superpathway of guanosine nucleotides de novo biosynthesis I|g__Bacteroides.s__Bacteroides_caccae	0.598	0.378 — 0.947	0.028	0.994
PWY-6700: queuosine biosynthesis I (de novo)|g__Bacteroides.s__Bacteroides_stercoris	0.54	0.309 — 0.942	0.03	0.994
PWY-7219: adenosine ribonucleotides de novo biosynthesis|g__Bacteroides.s__Bacteroides_stercoris	0.613	0.393 — 0.956	0.031	0.994

Supplementary References

S1.Inker LA, Eneanya ND, Coresh J, Tighiouart H, Wang D, Sang Y, Crews DC, Doria A, Estrella MM, Froissart M, Grams ME, Greene T, Grubb A, Gudnason V, Gutiérrez OM, Kalil R, Karger AB, Mauer M, Navis G, Nelson RG, Poggio ED, Rodby R, Rossing P, Rule AD, Selvin E, Seegmiller JC, Shlipak MG, Torres VE, Yang W, Ballew SH, Couture SJ, Powe NR, Levey AS; Chronic Kidney Disease Epidemiology Collaboration. New Creatinine- and Cystatin C-Based Equations to Estimate GFR without Race. N Engl J Med. 2021 Nov 4;385(19):1737-1749. doi: 10.1056/NEJMoa2102953.

STROBE Statement—Checklist of items that should be included in reports of ***cohort studies***

	Item No	Recommendation	Page No
**Title and abstract**	1	(*a*) Indicate the study's design with a commonly used term in the title or the abstract	1
		(*b*) Provide in the abstract an informative and balanced summary of what was done and what was found	3
**Introduction**			
Background/rationale	2	Explain the scientific background and rationale for the investigation being reported	5-6
Objectives	3	State specific objectives, including any prespecified hypotheses	5-6
**Methods**			
Study design	4	Present key elements of study design early in the paper	5-6
Setting	5	Describe the setting, locations, and relevant dates, including periods of recruitment, exposure, follow-up, and data collection	7-8
Participants	6	(*a*) Give the eligibility criteria, and the sources and methods of selection of participants. Describe methods of follow-up	7-8
		(*b*) For matched studies, give matching criteria and number of exposed and unexposed	
Variables	7	Clearly define all outcomes, exposures, predictors, potential confounders, and effect modifiers. Give diagnostic criteria, if applicable	8, suppl
Data sources/ measurement	8*	For each variable of interest, give sources of data and details of methods of assessment (measurement). Describe comparability of assessment methods if there is more than one group	7-8 + suppl
Bias	9	Describe any efforts to address potential sources of bias	N/A
Study size	10	Explain how the study size was arrived at	7
Quantitative variables	11	Explain how quantitative variables were handled in the analyses. If applicable, describe which groupings were chosen and why	8-9
Statistical methods	12	(*a*) Describe all statistical methods, including those used to control for confounding	
		(*b*) Describe any methods used to examine subgroups and interactions	
		(*c*) Explain how missing data were addressed	8-9
		(*d*) If applicable, explain how loss to follow-up was addressed	
		(*e*) Describe any sensitivity analyses	
**Results**			
Participants	13*	(a) Report numbers of individuals at each stage of study—eg numbers potentially eligible, examined for eligibility, confirmed eligible, included in the study, completing follow-up, and analysed	7,10, Table1
		(b) Give reasons for non-participation at each stage	
		(c) Consider use of a flow diagram	
Descriptive data	14*	(a) Give characteristics of study participants (eg demographic, clinical, social) and information on exposures and potential confounders	10, Table1

		(b) Indicate number of participants with missing data for each variable of interest<br>(c) Summarise follow-up time (eg, average and total amount)	
Outcome data	15*	Report numbers of outcome events or summary measures over time	10-11, Table1
Main results	16	(*a*) Give unadjusted estimates and, if applicable, confounder-adjusted estimates and their precision (eg, 95% confidence interval). Make clear which confounders were adjusted for and why they were included<br>(*b*) Report category boundaries when continuous variables were categorized<br>(*c*) If relevant, consider translating estimates of relative risk into absolute risk for a meaningful time period	10-11, Tables, Figures
Other analyses	17	Report other analyses done—eg analyses of subgroups and interactions, and sensitivity analyses	N/A
**Discussion**			
Key results	18	Summarise key results with reference to study objectives	12, 17
Limitations	19	Discuss limitations of the study, taking into account sources of potential bias or imprecision. Discuss both direction and magnitude of any potential bias	17
Interpretation	20	Give a cautious overall interpretation of results considering objectives, limitations, multiplicity of analyses, results from similar studies, and other relevant evidence	12-16
Generalisability	21	Discuss the generalisability (external validity) of the study results	12-17
**Other information**			
Funding	22	Give the source of funding and the role of the funders for the present study and, if applicable, for the original study on which the present article is based	18

*Give information separately for exposed and unexposed groups.

Note: An Explanation and Elaboration article discusses each checklist item and gives methodological background and published examples of transparent reporting. The STROBE checklist is best used in conjunction with this article (freely available on the Web sites of PLoS Medicine at http://www.plosmedicine.org/, Annals of Internal Medicine at http://www.annals.org/, and Epidemiology at http://www.epidem.com/). Information on the STROBE Initiative is available at http://www.strobe-statement.org.
